# Effects of the chemokine CXCL12 and combined internalization of its receptors CXCR4 and CXCR7 in human MCF-7 breast cancer cells

**DOI:** 10.1007/s00441-014-1823-y

**Published:** 2014-04-26

**Authors:** Kirsten Hattermann, Eric Holzenburg, Friederike Hans, Ralph Lucius, Janka Held-Feindt, Rolf Mentlein

**Affiliations:** 1 Department of Anatomy, University of Kiel, Olshausenstraße 40, 24098 Kiel, Germany; 2 Department of Neurosurgery, University Medical Center Schleswig-Holstein UKSH, Campus Kiel, 24105 Kiel, Germany

**Keywords:** Chemokine receptor, Breast cancer, Internalization, Signal transduction, Apoptosis

## Abstract

The chemokine CXCL12 (stromal cell-derived factor-1, SDF-1) and its receptor CXCR4 play a major role in tumor initiation, promotion, progression and metastasis, especially for breast cancer cells. Recently, CXCR7 has been identified as a second receptor for CXCL12; nevertheless, it also binds CXCL11 (interferon-inducible T cell α chemoattractant, I-TAC). However, little is known about the co-expression of the two receptors and their interactions. Quantitative reverse transcription plus the polymerase chain reaction has demonstrated that both receptors are frequently co-expressed in breast cancer cell lines, whereas other tumor cell lines often express only one of them. For interaction studies, we chose MCF-7 breast cancer cells, since they highly express CXCR4 and CXCR7 at the protein level but not CXCR3 (another target for CXCL11). Immunofluorescence and gold–labeling by light and electron microscopy, respectively, revealed that both receptors were localized at the cell surface in non-stimulated cells. After exposure to CXCL12 or CXCL11, the receptors were rapidly internalized alone or in close proximity. Stimulation with the CXCR4- or CXCR7-selective non-peptide antagonists AMD3100 and CCX733 resulted not only in single internalization but partly also in co-internalization of the two receptors. Furthermore, both chemokine ligands reduced staurosporine-induced apoptosis and caspase-3/7 activation; however, the selective inhibitors merely had partial inhibitory effects on these biological responses. Our findings suggest that CXCR4 and CXCR7 closely interact in breast cancer cells. Both are co-internalized, transduce signals and induce further biological effects partly independently of a selective stimulus or antagonist.

**Electronic supplementary material:**

The online version of this article (doi:10.1007/s00441-014-1823-y) contains supplementary material, which is available to authorized users.

## Introduction

Chemokines were initially discovered as small, 8- to 10-kDa chemotactic cytokines in the immune system; they attract leukocytes by interacting with their G-protein-coupled seven-transmembrane domain receptors. Later, chemokines and their receptors were shown to exhibit a much broader function in tissue development and homeostasis and in many pathological conditions (for a review, see Mentlein et al. 2013). In particular, they play decisive roles in tumor initiation, promotion, progression and metastasis (for a review, see Zlotnik et al. 2011). With respect to the last-mentioned, the chemokine CXCL12/SDF-1 (stromal cell-derived factor-1) and its receptor CXCR4 have attracted great interest as they direct CXCR4-expressing breast cancer cells to peripheral tissues such as lung, liver, lymph nodes and bone marrow where the ligand CXCL12 is constitutively produced (Müller et al. 2001). However, the interactions and regulations of chemokines and their receptors are highly complex and also in this case, the initially simple model has had to be modified (for a review, see Hattermann and Mentlein 2013). Namely, a second receptor for CXCL12 was recently de-orphanized and named CXCR7/RDC1 (Balabanian et al. 2005; Burns et al. 2006). In addition to CXCL12, CXCR7 binds with ten-fold lower affinity to CXCL11/I-TAC (interferon-inducible T cell α chemoattractant), which is also a ligand for CXCR3 (which is targeted also by CXCL9/Mig and CXCL10/IP-10).

At first, CXCR7 was thought to be merely a non-signaling decoy or scavenger receptor, since because of alterations in a conserved DRYLAIV-motif, it fails to couple to G-proteins (Naumann et al. 2010; Thelen and Thelen 2008) and does not cause Ca^2+^ mobilization upon activation, as is known for most chemokine receptors (Burns et al. 2006). However, recent reports show that CXCR7 signals alternatively through β-arrestin (Rajagopal et al. 2010) and thus directly mediates several cellular effects, in addition to its function in controlling extracellular CXCL12 levels. In cells expressing solely CXCR7, stimulation by CXCL12 or CXCL11 results in the phosphorylation of the kinases Erk (extracellular-signal regulated kinases (p42/p44)) and Akt, for example, in human leukocytes, vascular smooth muscle, rat glial, or human glioblastoma cells (Balabanian et al. 2005; Rajagopal et al. 2010; Hattermann et al. 2010; Ödemis et al. 2012). Regarding cellular responses, CXCR7 stimulation enhances cell survival of tumor cells, e.g., by reduction of temozolomide-induced apoptosis in glioblastoma cells or of cell adhesion (Burns et al. 2006; Miao et al. 2007; Hattermann et al. 2010, 2012).

However, some cell types, especially endothelial and distinct tumor cells, express the two CXCL12-receptors, CXCR4 and CXCR7, in combination (Miao et al. 2007; Heinrich et al. 2012) and they may regulate one another’s function. This can be studied by use of selective antagonists, by application of CXCL11 (if CXCR3 is absent), or by silencing/overexpression. Here, partially divergent reports exist. For example, CXCR7-activation can block CXCR4-driven transendothelial migration of lymphoma cells (Zabel et al. 2009), can impair CXCL12-induced tube formation of endothelial progenitor cells (Yan et al. 2012), facilitate CXCR4-mediated neuronal migration (Sanchez-Alcaniz et al. 2011) and enhance CXCR4-promoted chemotaxis in vitro but decreases matrix degradation and invasion in vivo in CXCR4-overexpressing MTLn3 breast cancer cells (Herandez et al. 2011). Indeed, CXCR4-CXCR7 heterodimerization has been recently observed by biochemical methods in transfected HEK cells (Levoye et al. 2009; Décaillot et al. 2011) and the ligand CXCL12 might also homodimerize even at low concentrations (Ray et al. 2012a).

Thus, cellular responses to CXCL12 (and CXCL11) might depend on the relative expression of the two receptors or on the cell type. Therefore, more clarification of their interactions is urgently required. Since breast cancer cells are an important target, we examined the expression of CXCR4 and CXCR7 in various cell lines and selected MCF-7 cancer cells as a representative that expresses both receptors at comparable levels but not CXCR3. We investigated the localization of CXCR4 and CXCR7, before and after chemokine-stimulation of these cells, by fluorescence and electron microscopy and evaluated the influence of receptor-selective antagonists on CXCL12/CXCL11-mediated signal transduction and its effects.

## Materials and methods

### Peptides and inhibitors

Recombinant human chemokines and growth factors were from Pepro Tech (Rocky Hill, N.J., USA; CXCL11) or Immunotools (Friesoythe, Germany; CXCL12, epidermal growth factor). Staurosporine was purchased from Calbiochem (Merck-Calbiochem, Darmstadt, Germany). The non-peptide antagonist AMD3100 (= plerixafor, Mozobil, selective for CXCR4) was obtained from Sigma-Aldrich (St. Louis, Mo., USA) and CCX733 (selective for CXCR7) was a generous gift from Mark E.T. Penfold from ChemoCentryx (Mountain View, Calif., USA). Staurosporine and antagonists were dissolved in dimethylsulfoxide and diluted from these stock solutions.

### Cell cultures and stimulations

MCF-7 mamma carcinoma cells were obtained from the American Type Culture Collection (ATTC) via Cell Line Service, Eppelheim, Germany and cultured in Dulbecco’s modified Eagle’s medium (DMEM) supplemented with 10 % fetal calf serum (FCS) and 1 % penicillin/streptomycin (P/S; all from PAN Biotech, Aidenbach, Germany); other mamma carcinoma cell lines (T47D, BT549, MDA-MB231) were obtained and kept as described elsewhere (Stark et al. 2007). The glioma cell line U343 was obtained from the Deutsches Krebsforschungszentrum (Heidelberg, Germany; cf. Mentlein and Held-Feindt 2002), cultivated in 10 % DMEM + 10 % FCS + 1 % P/S; melanoma cells (LOX, Mel6, Mel Juso), small cell lung cancer cells (OH1, OH2 and SW2) and the neuroblastoma cell line SH-SY5Y were generously supplied by Prof. Dr. Udo Schumacher (Department of Anatomy, University of Hamburg, Germany) and characterized and cultivated as described (Thies et al. 2007; Lange et al. 2011). All cells were routinely checked for *Mycoplasma* contamination by 4′,6-diamidino-2-phenylindole (DAPI) staining and *Mycoplasma*-specific polymerase chain reaction (PCR; Minerva Biolabs, Berlin, Germany).

### Quantitative reverse transcription plus PCR

RNA was isolated with the Qiazol Lysis reagent (Qiagen, Hilden, Germany) and digested by DNase. cDNA was synthesized and real-time reverse transcription plus PCR (RT-PCR) was performed (Ludwig et al. 2005) by using the following TaqMan primer probes (Applied Biosystems, Foster City, Calif., USA): human glyceraldehyde 3-phosphate dehydrogenase (hGAPDH; Hs99999905_m1), hCXCL11 (Hs00171138_m1), hCXCL12 (Hs00171022_m1), hCXCR3 (Hs00171041_m1), hCXCR4 (Hs00607978_s1) and hCXCR7 (Hs00664172_s1). Cycles of thresholds (CT) were determined with an ABI PRISM 7000 sequence detection system and ∆CT values = CT_Gene of interest_ - CT_GAPDH_ were calculated. A ∆CT value of 3.33 corresponds to one magnitude lower gene expression compared with GAPDH. For each gene, logarithmic linear dependence of CT-values from the numbers of copies was verified by using various amounts of cDNA.

### Western blotting and enzyme-linked immunoassay

Western blot experiments were performed as previously described (Hattermann et al. 2008). CXCR7 was detected with anti-CXCR7 (ab 38089, rabbit, 1:500; Abcam, Cambridge, Mass., USA); re-blotting was performed with anti-caveolin-1 (N-20, rabbit, 1:200; Santa Cruz Biotechnology, Santa Cruz, Calif., USA). For analysis of kinase phosphorylation, blots were incubated with anti-phosphorylated Erk (pErk1/2; Thr202/Tyr204; 1:500; Cell Signaling Technology, Danvers, Mass., USA) and re-probed after stripping with methanol and 0.1 mol/l glycine/HCl buffer, pH 2.5, with anti-Erk2 (1:500; Santa Cruz).

Enzyme-linked immunoassay (ELISA) for CXCL12 was performed with culture supernatants and standards, with recombinant protein being applied to Nunc Maxisorb 48-well plates (Nunc, Roskilde, Denmark) for 2 h at room temperature. After removal of samples, wells were blocked with 1 % bovine serum albumin (BSA) and 0.02 % Tween in phosphate-buffered saline (PBS) for 1 h, washed three times with PBS at pH 7.4, incubated with anti-CXCL12 (1:500; anti-SDFα, rabbit polyclonal, ab9797, Abcam), washed with PBS (3×), incubated with horseradish-peroxidase-labeled anti-rabbit IgG (1:1000, raised in goats, sc2030, Santa Cruz Biotechnology), washed (3×) and finally exposed to 100 μl 3,3′,5,5′-tetramethylbenzidine substrate (Thermo Scientific Pierce, Waltham, Mass., USA), the reaction being stopped with 50 μl 0.5 M H_2_SO_4_. Extinction at 450 nm was measured against a reference wavelength (620 nm).

### Internalization experiments and fluorescence light and electron microscopy

Subconfluent cells were grown on poly-D-lysine-coated coverslips in DMEM plus 10 % FCS for 1 day, washed with PBS plus 0.5 % BSA and equilibrated twice (1 h each) with DMEM plus 0.5 % fatty-acid-free BSA (Sigma-Aldrich). The cells were then gradually cooled to 4 °C and exposed (at 4 °C) to primary antibodies (for fluorescence light microscopy: rabbit anti-CXCR4, 1:100, Imgenex IMG-125, San Diego, Calif., USA; for electron microscopy: goat anti-CXCR4, 1:100, Abcam ab1671; fluorescence light and electron microscopy: mouse anti-CXCR7, 1:100, Chemocentrxy 11G8) for 45 min at 4 °C. For secondary antibody controls, primary antibodies were omitted. After being washed, cells were then incubated in the same medium with secondary antibodies for 45 min at 4 °C. For light microscopy, Alexa-Fluor-labeled donkey anti-mouse IgG 488 and donkey anti-rabbit IgG 555 (1:800 each; Invitrogen, Carlsbad, Calif.) were applied as secondary antibodies. For electron microscopy, colloidal-gold-labeled donkey anti-goat IgG with 15-nm gold (Aurion, Wageningen, The Netherlands) and, after several rinses, goat anti-mouse IgG (British BioCell, Cardiff, UK) conjugated to 5-nm gold were used as secondary antibodies. After two washes, medium was replaced with fresh medium (4 °C) containing ligands or antagonists. Cells were then warmed to 37 °C for various times (or left at 4 °C as controls) and fixed with Zamboni’s fixative consisting of 4 % formaldehyde freshly prepared from paraformaldehyde with 17.5 % saturated picrinic acid in phosphate buffer for fluorescence microscopy or with 4 % formaldehyde freshly prepared from paraformaldehyde and 0.5 % glutaraldehyde in PBS for electron microscopy. For fluorescence light microscopy, fixed cells were washed with PBS, incubated with Alexa-Fluor-647-labeled wheat germ agglutinin (WGA, 1:200, 10 min; Invitrogen), counterstained with DAPI and inspected with a Zeiss Axiovert 200 M microscope with ApoTome. Measurements of cytosolic:surface ratio were performed as described in Supplementary Fig. 1. For electron microscopy, specimens were washed with PBS, exposed to 2 % osmium tetroxide for 30 min, dehydrated in series with increasing ethanol concentrations and embedded in Araldite. Ultrathin (60 nm) sections were cut, contrasted in saturated uranyl acetate for 2 min and viewed under a Zeiss 902 electron microscope at a primary magnification of ×50,000 (cf. Krisch et al. 1998).

### Apoptosis and caspase assays

Cell death assays were performed in DMEM plus 0.2 % FCS for the indicated times. For detection of nuclei with signs of apoptosis, namely nuclear fragmentation and/or chromatin condensation, cells were seeded on poly-D-lysine-coated coverslips, grown overnight and then treated with staurosporine, chemokines and antagonists for 24 h. After being rinsed with PBS, cells were fixed with ice-cold acetone/methanol (1:1; 10 min) and washed (3×) with PBS. Nuclei were stained with DAPI for 30 min (modified after Nicoletti et al. 1991). Damaged nuclei were evaluated and counted by a non-biased person.

Caspase-3/7 activity was measured after an 8-h exposure to toxic agents and chemokines by collecting adherent and non-adherent cells by centrifugation for 5 min at 1000*g*, washes with PBS and lysis in 100-200 μl 0.1 % Nonidet P40 (NP40), 10 mM dithiothreitol, 1 mM EDTA, 100 mM NaCl in 50 mM HEPES buffer, pH 7.4. After a freeze-thaw cycle (30 min at -70 °C) and protein determination of an aliquot by the Bradford assay, samples were adjusted to equal protein concentrations and volumes (50 μg protein/250 μl) and incubated with 250 μl 40 μM N-Ac-Asp-Glu-Val-Asp-AMC (Ac-DEVD-AMC; Bachem, Torrance, Calif., USA; AMC, 7-amino-4-methylcoumarine) in lysis buffer at 37 °C. Fluorescence of liberated AMC was determined after various times (λ_Ex_ = 360 nm; λ_Em_ = 460 nm) and the maximum value was set as 100 %. Controls were performed with identical incubations in the presence of 0.6 nM N-Ac-Asp-Glu-Val-Asp-aldehyde (Ac-DEVD-CHO), a potent and selective inhibitor of caspases-3/7 and subtracted from the values.

### Statistical analysis

Values are given as means ± standard deviations (SD). Statistical significance was analyzed by a paired two-tailed Student’s *t*-test; *P < 0.05, **P < 0.01, ***P < 0.001.

## Results

### CXCR4 and CXCR7 are both expressed and produced by breast cancer cells

**Fig. 1:**
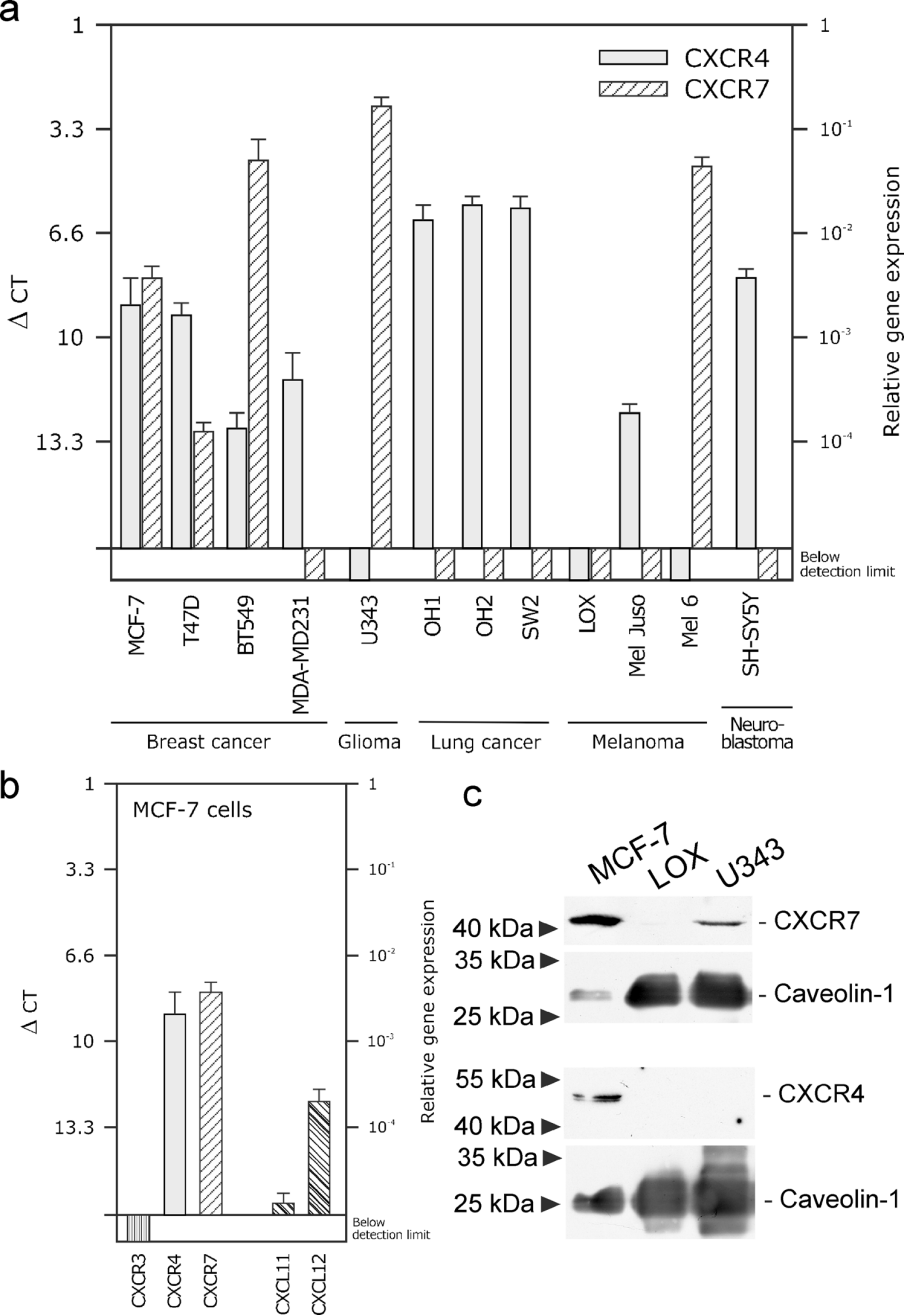
Expression of chemokine receptors in MCF-7 and other tumor cells of various origin. **a** Transcription of chemokine receptors CXCR4 and CXCR7 in various types of tumor cells was determined by quantitative reverse transcription plus the polymerase chain reaction (RT-PCR). ∆CT values relatve to glyceraldehyde 3-phosphate dehydrogenase (GAPDH) are given (*n* = 3 ± S.D.); ∆CT = 3.33 corresponds to one magnitude. Breast cancer cell lines show various levels of CXCR4 and CXCR7 transcription; mostly, both receptors are co-expressed. In contrast to mamma carcinoma cells, glioma cells (*U343*) exhibit a preferential CXCR7 expression (for more data, see Hattermann et al. 2010), whereas small cell lung cancer cells (*OH1*, *OH2*, *SW2*) and the neuroblastoma cell line (*SH-SY5Y*) transcribe only CXCR4. In melanoma cell lines, both receptors are either not detectable (*LOX*) or singularly transcribed (in *Mel Juso*, only CXCR4; in *Mel6*, only CXCR7). **b** Transcription of chemokine ligands and receptors in MCF-7 breast cancer cells as determined by quantitative RT-PCR (qRT-PCR; see **a**). CXCR4 and CXCR7 are both transcribed but not CXCR3 (but primers/probe yield a signal with T cells as positive controls, not shown). Moreover, the CXCR4/7 ligand CXCL12 is expressed at a low level, whereas the CXCR3/7 ligand CXCL11 is just above the detection limit. **c** Western blots of chemokine receptors CXCR4 and CXCR7 in membrane fractions of various types of human tumor cells clearly reveal a dual production of CXCR4 and CXCR7 in MCF-7 breast cancer cells, a single expression of CXCR7 in U343 glioma cells and an absence of both receptors in LOX melanoma cells, as also found by qRT-PCR (caveolin-1: loading control)

In an initial experiment, we investigated the expression of the chemokine receptors CXCR7 and CXCR4 in various breast cancer and other tumor cell lines by quantitative RT-PCR and Western blots (Fig. 1). Whereas the investigated breast cancer cells mostly transcribed (at different levels) both receptors, other tumor cell lines tested displayed a more restricted or no detectable expression (Fig. 1a). CXCR7 is prominent in human glioma cell lines such as U343 or A772 cells, as previously reported (Hattermann et al. 2010; with more examples). In contrast, the small cell lung cancer cell lines OH1, OH2 and SW2 transcribed all CXCR4 but were CXCR7-negative. Among melanoma cell lines, some were negative for both receptors; others showed expression of the one or other receptor. The frequently used neuroblastoma cell line SH-SY5Y showed a relative high CXCR4 mRNA level but was CXCR7-negative. Hence, many but not all types of cancer cells express CXCR4 or CXCR7 in various combinations.

**Fig. 2:**
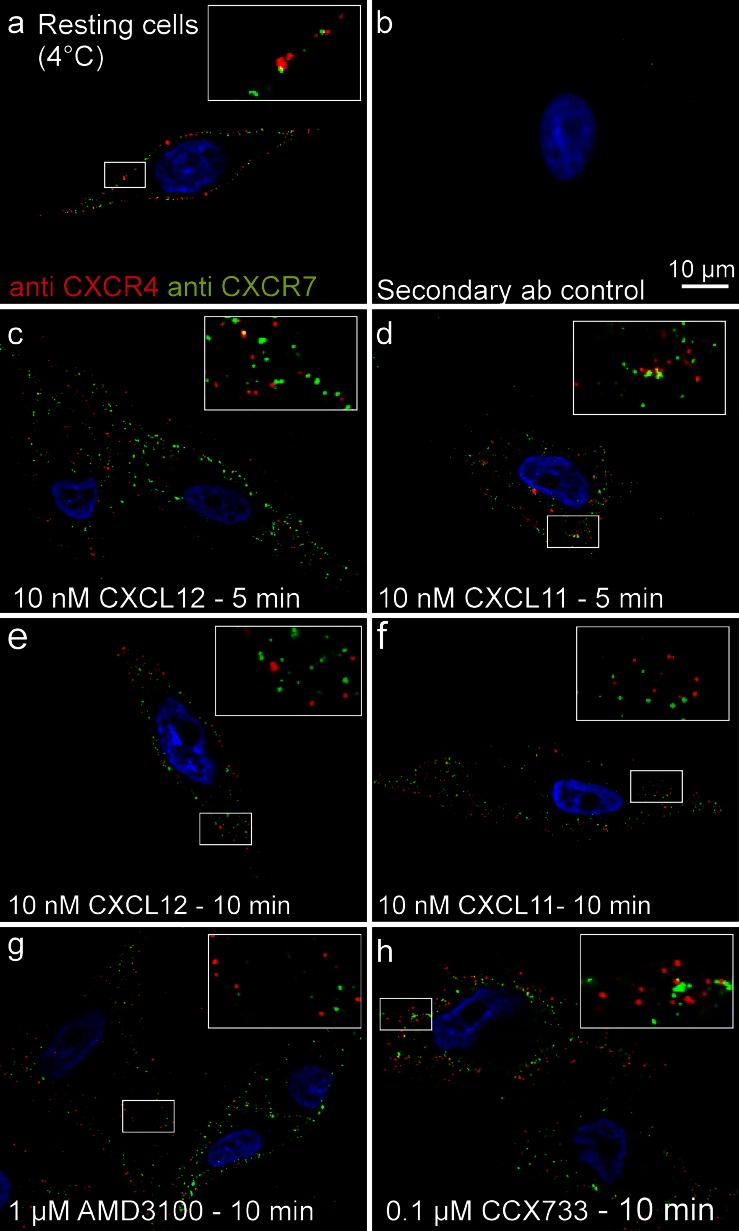
Internalization of chemokine receptors CXCR4 and CXCR7 into MCF-7 cells upon stimulation with various ligands, as visualized by immuno-fluorescence light microscopy. **a** Chemokine receptors were immuno-labeled at 4 °C with *red* (CXCR4) and *green* (CXCR7) fluorescent (secondary) antibodies in resting cells. Without stimulation, receptors were scattered alone or lay in close proximity at the cell surface. **b** For secondary antibody controls, primary antibodies were omitted. **c-h** Internalization was induced by stimulation with various ligands at various times at 37 °C. After exposure to chemokines CXCL12 (10 nM; **c**, **e**) or CXCL11 (10 nM; **d**, **f**) or to non-peptide receptor-selective antagonists AMD3100 (1 μM; **g**) or CCX733 (0.1 μM; **h**) receptors were rapidly internalized mostly or partly together (see also *inserts*)

Since MCF-7 cells are established mamma carcinoma cells and transcribe both chemokine receptors at comparably high levels, we selected this cell line in order to study their interaction in an exemplary cell line. Receptor production at the protein level was verified by Western blot (Fig. 1c) and by immunocytochemistry (Fig. 2). Quantitative RT-PCR confirmed the absence of CXCR3, a competing receptor for CXCL11 and demonstrated a moderate production of the ligands CXCL11 and CXCL12 (Fig. 1b). To evaluate self-stimulation effects, endogenously produced CXCL12 was determined by ELISA of culture supernatants. After 24 h in culture, 500,000 MCF-7 cells produced 187 ± 7 ng CXCL12 (in 1 ml medium, *n* = 3 ± S.D.) yielding a concentration of about 23 ± 1 nM. However, in most experiments, much lower cell numbers and incubations times were employed ensuring a much lower concentration during stimulations (less than 0.1 nM). Furthermore, cells were carefully washed prior to experiments in order to exclude self-stimulation and to ensure the recovery of internalized receptors.

### CXCR4 and CXCR7 are jointly internalized upon ligand or antagonist stimulation

**Fig. 3:**
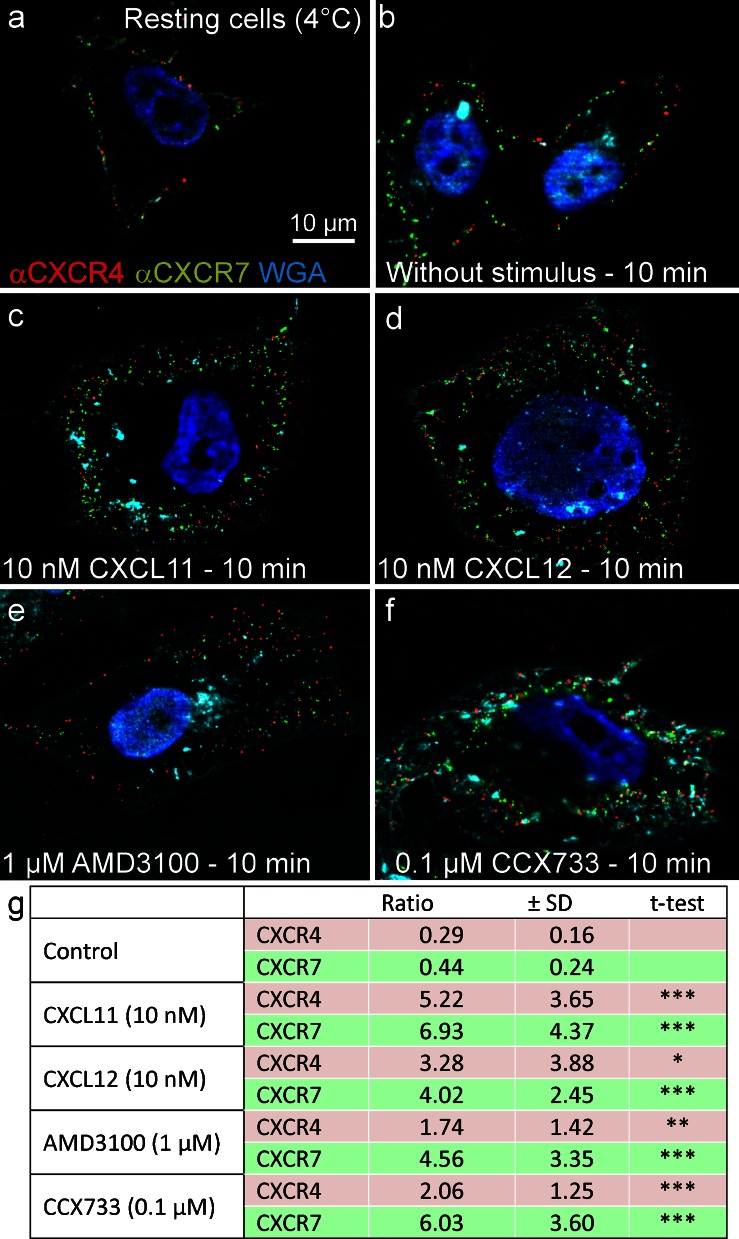
Measurement of CXCR4 and CXCR7 localization. **a-f** Chemokine receptors were immuno-stained and internalization was induced by stimulation at 37 °C (cf. Fig. 2). Membranes were stained with Alexa-Fluor-647-labeled wheat germ agglutinin (WGA, here displayed in *cyan*). **g** Based on the membrane signal, the intensity of CXCR4 and CXCR7 signals was measured, yielding the cytosolic:surface ratio (means ± SD). Unstimulated cells yielded low ratios (most receptors were on the surface), whereas both chemokines and synthetic antagonists yielded high ratios (most receptors were found intracellularly). For each stimulus, at least 10 cells were analyzed from two independent experiments (the quantification method is depicted in Supplementary Fig. 1)

**Fig. 4:**
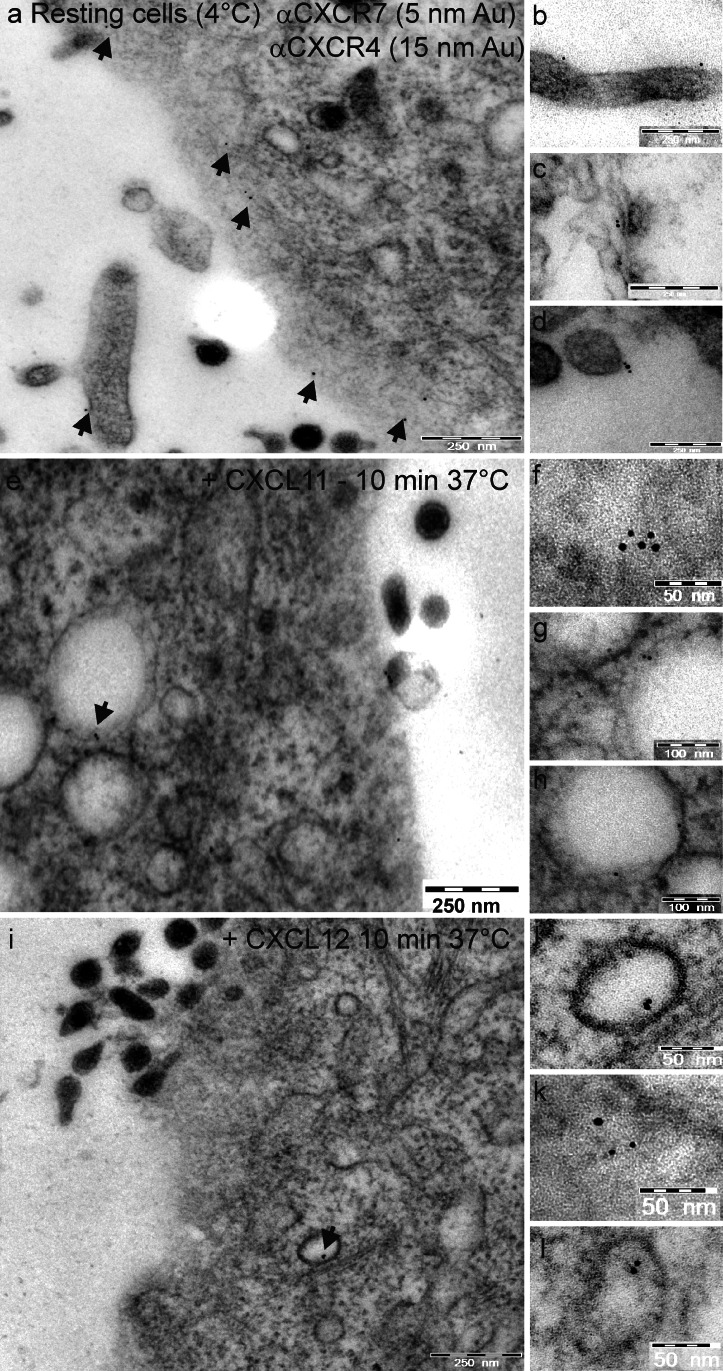
Internalization of chemokine receptors CXCR4 and CXCR7 in MCF-7 cells upon stimulation with various ligands visualized by immuno-gold electron microscopy. CXCR4 was immuno-labeled by 15-nm gold particles and CXCR7 by smaller 5-nm gold particles at 4 °C. Internalization was followed under stimulation with various ligands at various times at 37 °C, as correspondingly described in Fig. 2 (*arrowheads* gold particles). **a–d** On resting cells, both labels were found on the cell surface mostly alone as single dots but also sometimes in close proximity as clusters of small and large dots. **e–l** Upon ligand-induced stimulation, receptors were internalized and found in intracellular vesicles. Here, they frequently accumulated in groups of dots of one or mixed sizes. This co-internalization was observed either with CXCL11 as the CXCR7-selective ligand (**e-h**) or with CXCL12 as the ligand for both receptors (**i-l**). To improve the visualization of the gold particles, sections were only weakly exposed to osmium tetroxide and lead citrate

On living cells, receptors can be labeled at 4 °C by antibodies directed against their extracellular domains and visualized by use of differently fluorescent-labeled or gold-labeled secondary antibodies by using fluorescence or electron microscopy, respectively. On resting cells, both receptors can be readily stained at the cell surface by fluorescence (Figs. 2 and 3) and electron (Fig. 4) immunocytochemistry. As seen in the merged images of fluorescence by light microscopy or directly with the gold particles by electron microscopy, receptors are located separately and in close association.

After exposure to 37 °C, both receptors were rapidly internalized in the presence of ligands or antagonists and finally found in intracellular vesicles (Figs. 2, 3, 4). As seen best in immunofluorescence, CXCL12 stimulation initially resulted in a mostly separate internalization of both receptors (5 min, Fig. 2c, insert) as detected by separate red and green dots and a lower frequency of yellow (merged fluorescence) dots. However, after 10 min, nearly all dots were intracellularly located (Fig. 2e). With CXCL11, which binds only to CXCR7, similar internalization kinetics were observed but co-internalization of the two receptors was somewhat delayed; namely, after 5 min, red and green dots were located separately but at 10 min, the images were mostly similar to those of CXCL12 (Fig. 2d, f).

Semi-quantification of receptor internalization was achieved by labeling the glycocalyx of the cell surface with WGA (a lectin that binds to sialic acid and *N*-acetyl-D-glucosamine) and determining the ratio of surface versus intracellular chemokine receptor fluorescence (Fig. 3; see Supplementary Fig. 1 for method).

Exposure of the receptor-labeled cells to selective non-peptide antagonists AMD3100 or CCX733 (see Discussion) also yielded internalization of the receptors (Figs. 2g, h, 3e, f). Again, apart from receptor-selective internalization, namely CXCR4 by AMD3100 and CXCR7 by CCX733, co-internalization of the two receptors by their respective selective antagonist also occurred.

These results show that, on resting cells, both receptors are located at the cell surface alone or in close proximity. Upon stimulation with CXCL12 or CXCL11, rapid internalization of both chemokine receptors occurs. Furthermore, CXCR4 is also internalized by the CXCR7-ligand CXCL11 suggesting an interaction of both receptors. Not only exposure to ligands but also exposure to selective antagonists results in rapid receptor internalization. In addition to morphological connections, we next wanted to determine whether signal transductions and biological effects of both receptors were interconnected.

### Signal transduction is initiated through both receptors by CXCL12 stimulation

**Fig. 5:**
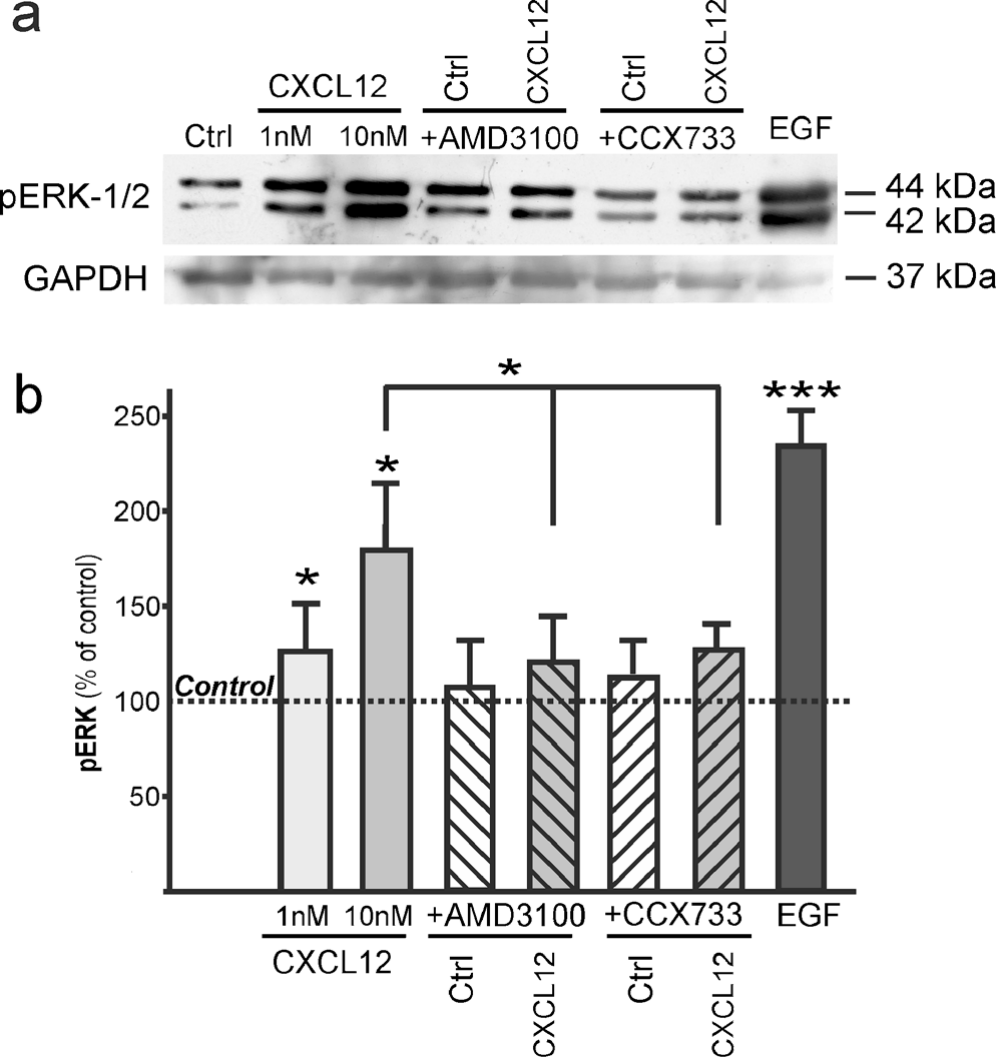
Phosphorylation of the extracellular-signal-regulated kinases Erk1/2 (p42/p44) in MCF-7 cells upon stimulation with CXCL12 in the absence and presence of CXCR4- and CXCR7-specific antagonists (*pErk* phosphorylated Erk, *Ctrl* control with antagonists alone). **a**, **b** Cells were stimulated for 15 min at 37 °C with ligand (1 or 10 nM), antagonists (AMD3100, 10 μM; CCX733, 0.1 μM), combinations, or a positive control (10 ng/ml epidermal growth factor, *EGF*), lysed and analyzed by Western blots probed with antibodies for the phosphorylated kinases and GAPDH (or re-probed for Erk2, not shown) to ensure equal loading. Example of several independent stimulations (*top*) and means ± SD of densitometry of *n* = 5 experiments (*bottom*). Stimulations were performed in serum-free media plus 0.1 % bovine serum albumin on washed and 1-h-pre-equilibrated cells. The inhibitors were added from stock solutions in dimethylsulfoxide (DMSO, 0.1 % final concentrations) at 1 h prior to the experiment; a corresponding amount of DMSO was added to all other cultures

When MCF-7 cells were stimulated with CXCL12, we observed phosphorylation of the kinases Erk1/2 in Western blots with a phosphorylation-specific antibody (Fig. 5). Phosphorylation could be significantly diminished by the receptor-selective non-peptide antagonists AMD3100 (for CXCR4) and by CCX733 (for CXCR7); each inhibitor induced no effects on its own (Fig. 5). These experiments show that stimulation by CXCL12 induces, in MCF-7 cells, a signal transduction that can be inhibited by receptor-selective non-peptide antagonists.

### CXCR4 and CXCR7 mediate apoptosis resistance

**Fig. 6:**
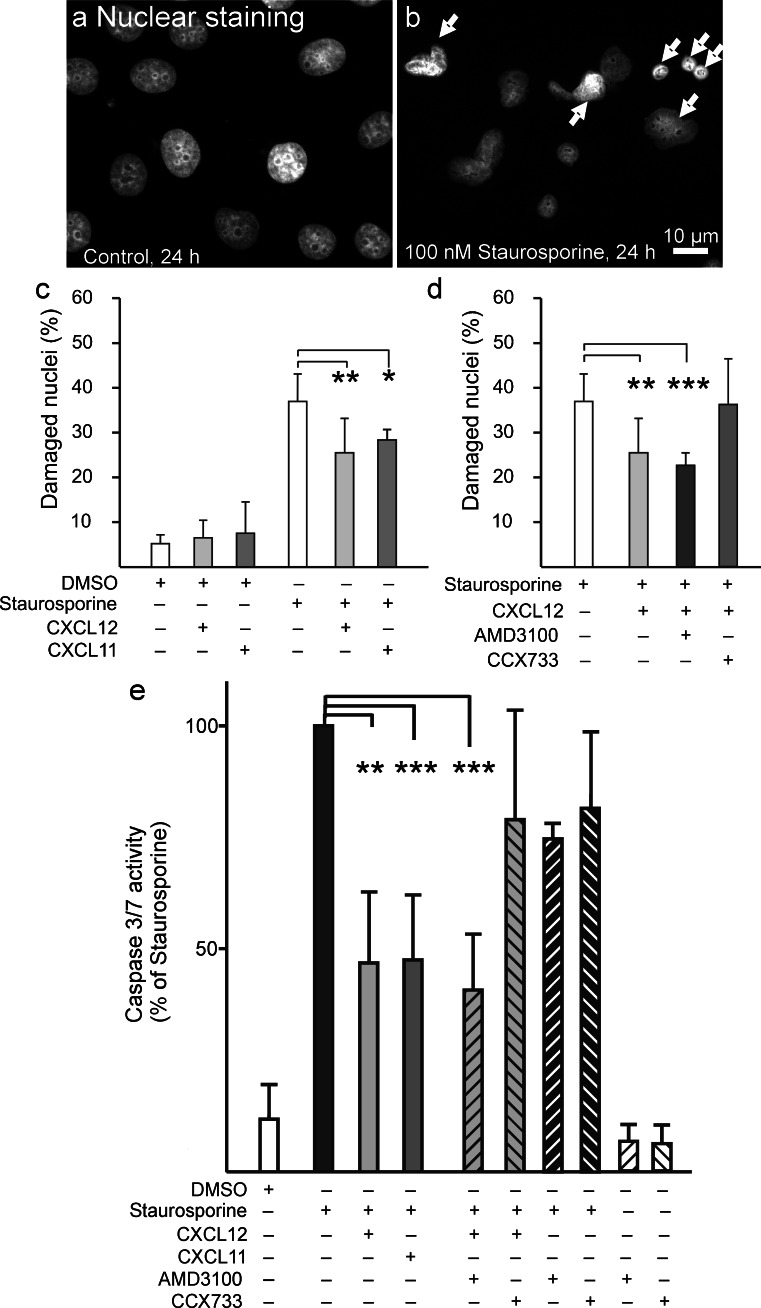
Inhibition of staurosporine-induced apoptosis and caspase-3/7 activation by CXCL11 and CXCL12 in MCF-7 cells and influence of selective CXCR4 and CXCR7 inhibitors. Apoptosis was induced by 100 nM staurosporine in DMEM + 0.2 % FCS and analyzed by quantification of apoptotic nuclei after 24 h or by measurement of caspase-3/7-activity after 8 h. **a**, **b** Visualisation of normal nuclei (**a**) and nuclei with signs of apoptosis (**b**, *arrows* nuclei damaged by fragmentation and/or chromatin condensation). **c** Both chemokine ligands, namely 5 nM CXCL11 and 1 nM CXCL12, significantly reduced staurosporine-induced apoptosis. **d** This anti-apoptotic effect could be reversed by co-incubation with the CXCR7-selective antagonist CCX733 (0.1 μM) but not significantly by CXCR4-selective antagonist AMD3100 (5 μM). Both antagonists had no inhibitory effects on their own (not shown). Means of triplicate counting of several inspection areas from *n* = 4 ± SD individual stimulations by an unbiased person. **e** Corroborating the morphological results, both chemokine ligands, namely 5 nM CXCL11 and 1 nM CXCL12, significantly reduced staurosporine-induced caspase-3/7 activation. Again, the antagonist CCX733 (0.1 μM) but not AMD3100 (5 μM), reduced this anti-apoptotic effect. Means of duplicate measurements from *n* = 3 ± SD individual stimulations (*P < 0.05, **P < 0.01, ***P < 0.001). Antagonists showed neither effect on basal or staurosporine-induced capsase activities. Staurosporine and antagonists were added from stock solutions in DMSO yielding a final concentration of 0.5 %; therefore, corresponding amounts of DMSO were added to all other incubations

To evaluate whether selective pharmacological inhibition also triggers combined biological effects, we measured anti-apoptotic effects previously preferentially attributed to CXCR7 (Hattermann et al. 2010). Apoptosis could be induced in MCF-7 cells by treatment with staurosporine. After 24 h, about 40 % of the cells showed apoptotic nuclei at a concentration of 100 nM staurosporine (Fig. 6b, Supplementary Fig. 2). Co-incubation with both chemokines, CXCL11 or CXCL12, reduced apoptotic nuclei about 25 % (Fig. 6c). The CXCL12-induced reduction of apoptosis could be inhibited by the CXCR7-selective antagonist CCX733 but not by the CXCR4-selective antagonist AM3100 (Fig. 6d). To corroborate these morphological findings by biochemical measurements, the activity of the effector caspases-3/7 was determined. After an 8-h exposure to 100 nM staurosporine, caspase-3/7 activity was more than 10-fold higher than that of controls (Fig. 6e). Again, CXCL11 or CXCL12 efficiently reduced this increase by 40-50 %. Whereas the CXCR4-antagonist AMD3100 did not influence CXCL12-induced apoptosis induction, the CXCR7-antagonist reduced the chemokine effect.

These experiments show that CXCL11 and CXCL12 both reduce chemically induced apoptosis. However, this anti-apoptotic effect is better inhibited by the CXCR7 antagonist.

## Discussion

Among chemokine receptors, CXCR4 has been found to be most widely expressed in many types of tumors and has been shown to be involved in tumor cell invasion, metastasis, survival and proliferation (Müller et al. 2001; for a review, see Zlotnik et al. 2011). Reports on the occurrence and biological role of the newly discovered second receptor for the CXCR4-ligand CXCL12, namely CXCR7, in tumors are emerging (Burns et al. 2006; Miao et al. 2007; Wang et al. 2008; Hattermann et al. 2010; for a review, see Hattermann and Mentlein 2013). Few but often conflicting results have been published on the interaction of CXCR4 and CXCR7. Knowledge of the interactions between both CXCL12 receptors is of particular importance in tumor biology, because they are co-expressed on many tumor cells, including breast cancer cells and on tumor endothelial cells and tumor-associated macrophages (Miao et al. 2007; Hattermann et al. 2010; Heinrich et al. 2012). Since MCF-7 cells express both receptors at comparable levels, we used them as a model to investigate the internalization of CXCR4 and CXCR7 and to elucidate the biological effects of the common ligand CXCL12 or the specific CXCR7-ligand CXCL11 and during the application of selective antagonists.

By immunolabeling of intact cells, we were able to localize both receptors clearly at the cell surface by fluorescence and electron microscopy. This partly contrasts with a few reports that are based on immunocytochemistry of fixed cells and that claim a preferential intercellular localization of CXCR7 (Luker et al. 2010; Ray et al. 2012b). However, localization of CXCR7 has also been demonstrated at the cell surface (Kumar et al. 2012; Hattermann et al. 2012). Pre-labeling of resting cells (without internalization, at 4 °C) with antibodies has now clearly established the cell surface localization of both receptors. Culture conditions, e.g., serum content or pre-stimulation, might greatly influence localization at the cell surface or intracellularly. By means of immuno-fluorescence and electron-microscopy, we were also able to show that, upon stimulation, both receptors are internalized rapidly and in close contact. Our findings corroborate experiments with transfected HEK-293 cells investigated by bioluminescence resonance energy transfer, which revealed the heterodimerization of overexpressed CXCR4 and CXCR7 receptors (Levoye et al. 2009). Interestingly, stimulation with receptor-specific agonists and even with non-peptide antagonists results in pronounced co-internalization of the receptors.

Chemokine receptors are composed of about 340-370 amino acids with a short acidic N-terminal extracellular domain, seven helical transmembrane domains plus three intracellular and three extracellular hydrophilic loops and an intracellular C-terminus containing serine and threonine residues that act as phosphorylation sites during receptor regulation (Allen et al. 2007). Receptor desensitization and internalization is achieved by agonist-dependent phosphorylation of the C-terminal tail, thereby promoting the binding of β-arrestins and internalization through clathrin-coated pits or lipid rafts/caveolae (Canals et al. 2012). Stimulation of CXCR4 by CXCL12 is known to activate heterotrimeric G-proteins resulting in the dissociation of the Gα and Gβγ subunits, which then activate downstream effectors (Busilo and Benovic 2007). A DRY-motif (DRYLAIV) in the second intracellular loop is thought to be essential for this G-protein-mediated signaling. In contrast to CXCR4, CXCR7 lacks this domain and was initially regarded as a decoy receptor that regulates CXCL12 levels (Naumann et al. 2010). Nevertheless, recent studies have shown that CXCR7 can signal via an alternative pathway, namely via β-arrestins that are also involved in the internalization of G-protein-coupled receptors (Rajagopal et al. 2010; Ödemis et al. 2012; Luttrell et al. 2010). However, the issue of CXCR7 signaling is still controversial.

**Fig. 7:**
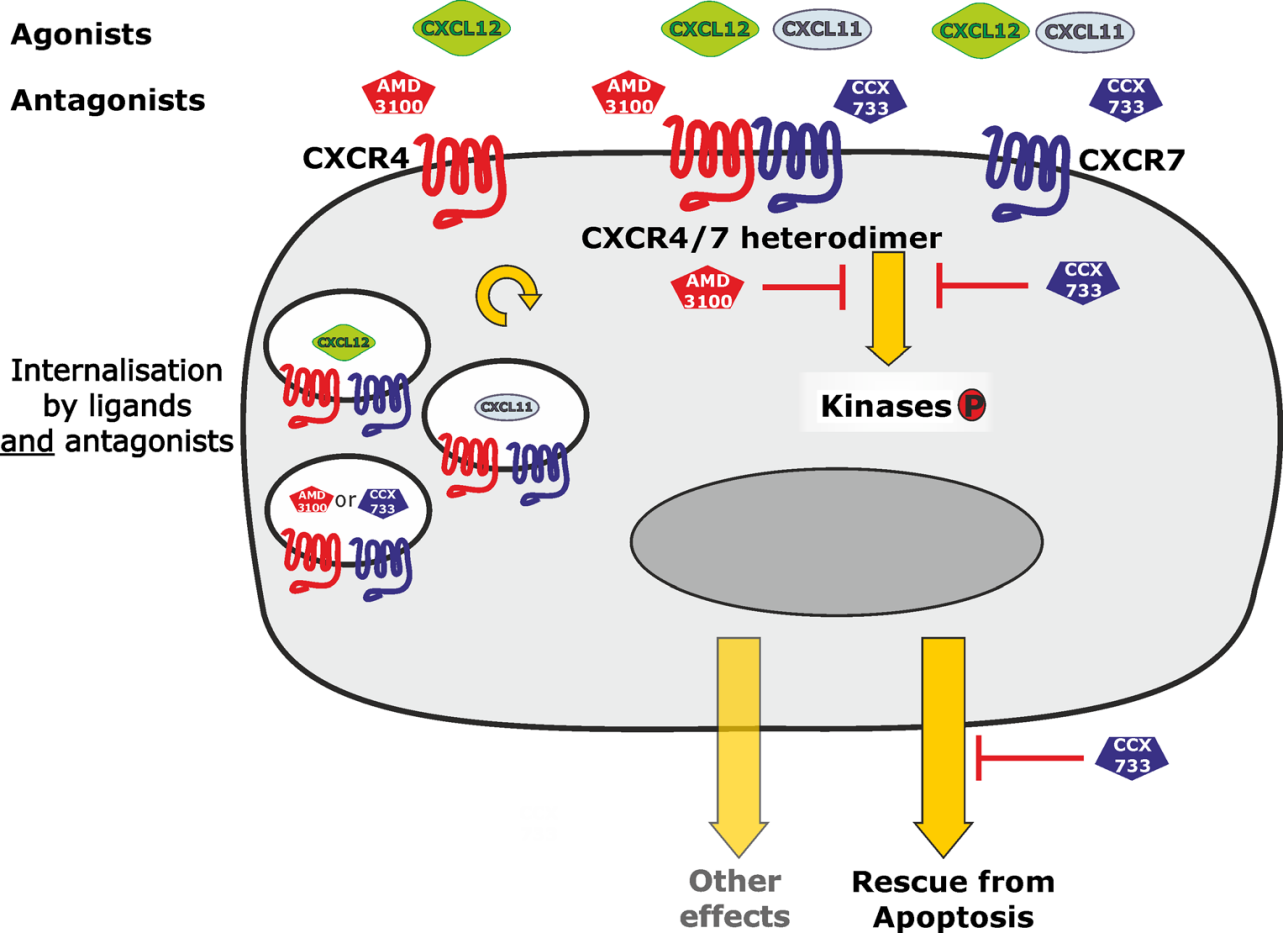
Representation of CXCR4 and CXCR7 in human breast cancer cells upon stimulation with selective agonists or antagonists

Based on our observations and previously reported coupled effects (Levoye et al. 2009; Ödemis et al. 2012), CXCR4-CXCR7 heterodimers appear to be more distinct functional units than the receptors alone (Fig. 7). Dual activating effects on proliferation have been recently described in CXCR4-CXCR7-coexpressing pancreatic cancer (Heinrich et al. 2012) or in Jurkat (Kumar et al. 2012) cells. As shown here, CXCL12-induced kinase phosphorylation in MCF-7 cells can be inhibited by selective agonists of one or the other receptor. In contrast, anti-apoptotic effects that are mediated by CXCL12 and CXCL11 alone are mainly antagonized by CCX733, the selective CXCR7 antagonist and not by AMD3100 antagonizing CXCR4. However, AMD3100 has been reported to act not solely as a simple CXCR4 antagonist (Kalatskaya et al. 2009). As measured by bioluminescence resonance energy transfer, AMD3100 also increases CXCL12 binding to CXCR7 and CXCL12-induced conformational rearrangements in the receptor dimer. Moreover, small increases in the potency of CXCL12-induced arrestin recruitment to CXCR7 by AMD3100 have been observed (Kalatskaya et al. 2009).

Thus, heterodimerization influences not only receptor internalization but also signal transduction and biological effects. Moreover, the pharmacological profiles of specific inhibitors are changed, as shown in our study.

Overall, the CXCL12-CXCR4-CXCR7-axis is highly complex. The bioavailability of CXCL12 (and CXCL11) is controlled by ligand dimerization (Ray et al. 2012a), by binding to heparan sulfate glycosaminoglycans on the cell surface (Allen et al. 2007), by proteolytic degradation (Ludwig et al. 2002; Mentlein 2004) and by receptor-mediated internalization (as shown here). Biological effects depend on the receptor subtype expressed by the particular cell type and on receptor interactions, if both subtypes are expressed.

## Electronic supplementary material

Below is the link to the electronic supplementary material.

Supplementary Fig. 1 Principle of quantification of CXCR4 and CXCR7 localization and internalization. Digital micrographs of CXCR4 (*red*) and CXCR7 (*green*) immuno-stained and wheat germ agglutinin (*WGA*)-stained (*cyan*) cells were taken and the channels were split into red/green/blue. The cyan-blue channel revealing membrane staining was used to define a surface and a cytosolic mask (with Corel Photo Paint). The masks were transferred to the green and red (not shown) channels. The mask contents were copied and black/white-inverted new files were produced (yielding *black dots* for each label). These signals were quantified with densitometry software (PCBAS) and the ratio of cytosolic:surface localization was calculated (mean ± SD). (JPEG 48 kb)

High resolution image (TIFF 3022 kb)

Supplementary Fig. 2 Induction of apoptosis in MCF-7 cells by staurosporine as determined by quantification of apoptotic nuclei (cf. Fig. 5). *Top* Time dependency. Maximal apoptosis is observed after 20-24 h. *Bottom* Dose dependency after 24 h; significant apoptosis occurs with 50 nM staurosporine and is maximal with 500 nM staurosporine. (JPEG 27 kb)

High resolution image (TIFF 80 kb)

## Abbreviations

AMC: 7-Amino-4-methylcoumarine
BSA: Bovine serum albumin
CXCL11 or I-TAC: Interferon-inducible T cell α chemoattractant
CXCL12 or SDF-1: Stromal cell-derived factor-1
DAPI: 4′,6-Diamidino-2-phenylindole
DMEM: Dulbecco’s modified Eagle’s medium
DMSO: Dimethylsulfoxide
EGF: Epidermal growth factor
ELISA: Enzyme-linked immunoassay
Erk: Extracellular-signal-regulated kinases (p42/p44)
FCS: Fetal calf serum
GAPDH: Glyceraldehyde 3-phosphate dehydrogenase
PBS: Phosphate-buffered saline
P/S: Penicillin/streptomycin
RT-PCR: Reverse transcription plus the polymerase chain reaction
WGA: Wheat germ agglutinin
