# Supplementary figures and images for: Effects of the chemokine CXCL12 and combined internalization of its receptors CXCR4 and CXCR7 in human MCF-7 breast cancer cells
Source: Cell Tissue Res. 2014 Apr 26;357(1):253–66. doi: 10.1007/s00441-014-1823-y (PMC4077318; doi:10.1007/s00441-014-1823-y)

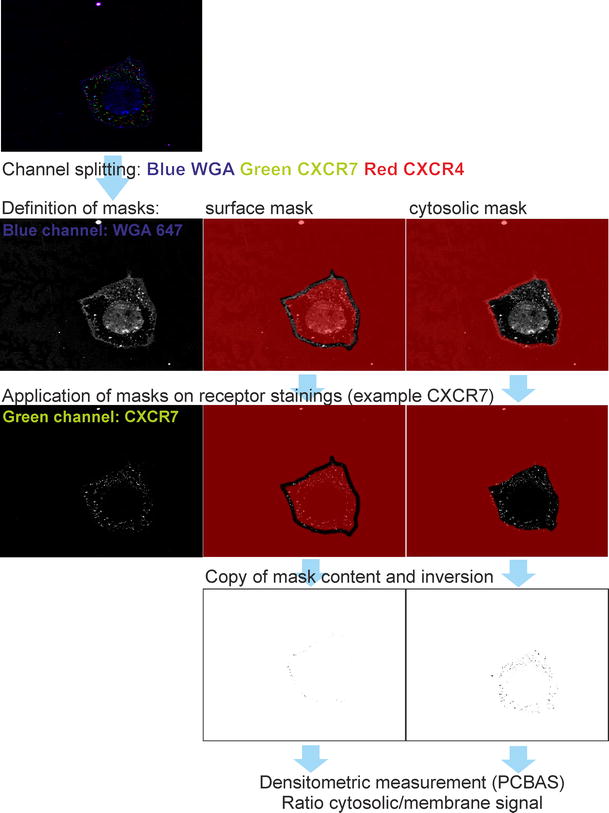

Supplement: Supplementary file 1 — Principle of quantification of CXCR4 and CXCR7 localization and internalization. Digital micrographs of CXCR4 (red) and CXCR7 (green) immuno-stained and wheat germ agglutinin (WGA)-stained (cyan) cells were taken and the channels were split into red/green/blue. The cyan-blue channel revealing membrane staining was used to define a surface and a cytosolic mask (with Corel Photo Paint). The masks were transferred to the green and red (not shown) channels. The mask contents were copied and black/white-inverted new files were produced (yielding black dots for each label). These signals were quantified with densitometry software (PCBAS) and the ratio of cytosolic:surface localization was calculated (mean ± SD). (JPEG 48 kb) [file 441_2014_1823_Fig8_ESM.jpg]

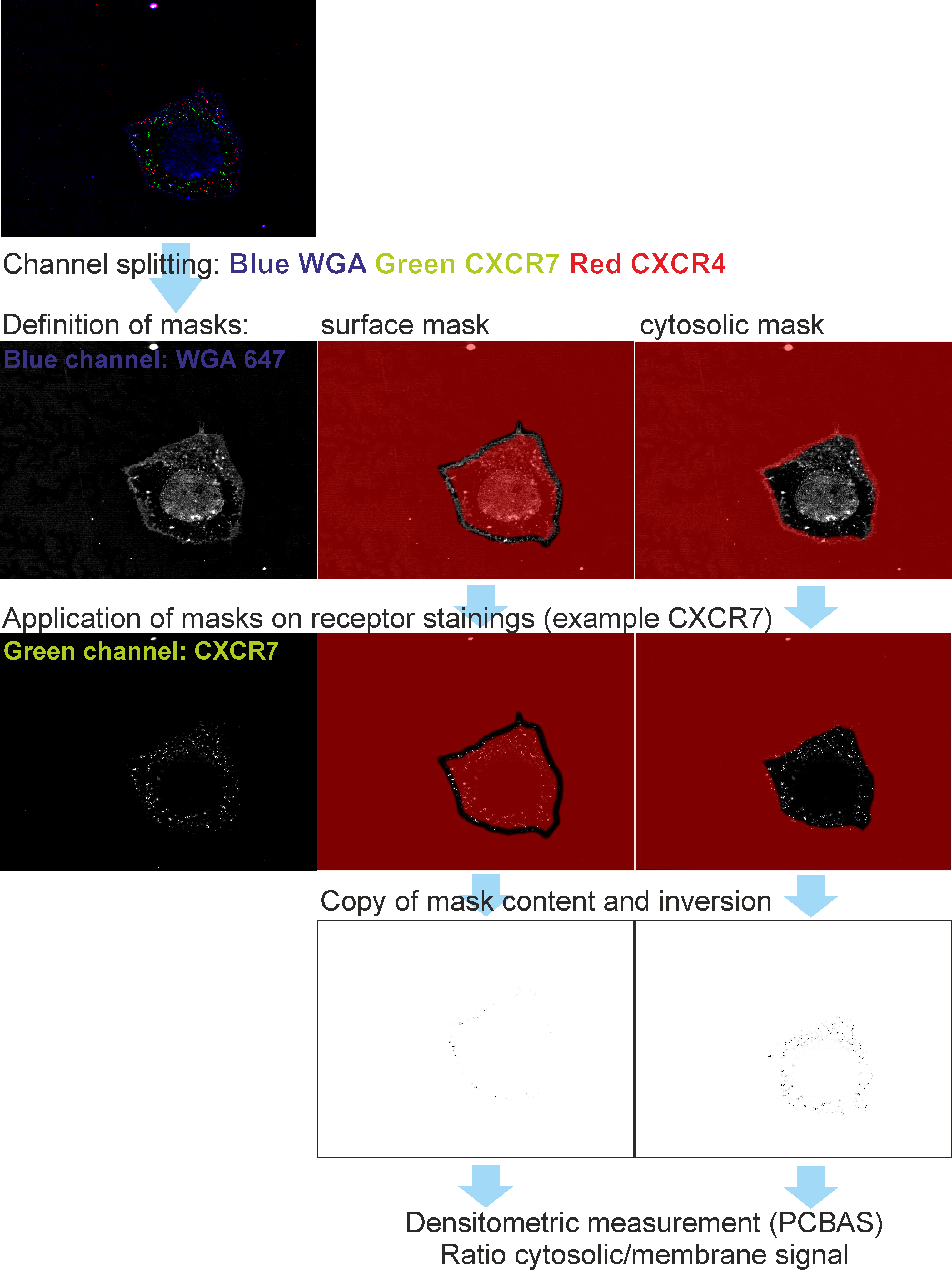

Supplement: Supplementary file 2 — High resolution image (TIFF 3022 kb) [file 441_2014_1823_MOESM1_ESM.tif]

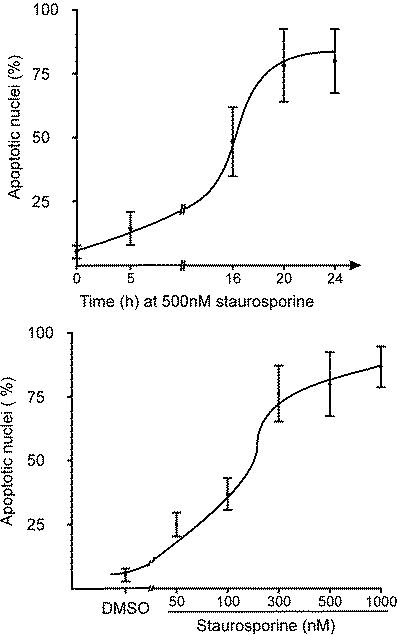

Supplement: Supplementary file 3 — Induction of apoptosis in MCF-7 cells by staurosporine as determined by quantification of apoptotic nuclei (cf. Fig. 5). Top Time dependency. Maximal apoptosis is observed after 20-24 h. Bottom Dose dependency after 24 h; significant apoptosis occurs with 50 nM staurosporine and is maximal with 500 nM staurosporine. (JPEG 27 kb) [file 441_2014_1823_Fig9_ESM.jpg]
